# Increased Interleukin-6 Levels in Responders with Treatment-Resistant Depression After Bright Light Therapy

**DOI:** 10.3390/biom15020295

**Published:** 2025-02-16

**Authors:** Biljana Kosanovic Rajacic, Marina Sagud, Drazen Begic, Matea Nikolac Perkovic, Ana Kozmar, Dunja Rogic, Alma Mihaljevic Peles, Marija Bozicevic, Nela Pivac

**Affiliations:** 1 Department for Psychiatry and Psychological Medicine, University Hospital Centre Zagreb, 10000 Zagreb, Croatia; bkosanov@gmail.com (B.K.R.); marinasagud@mail.com (M.S.); drazen.begic@mef.hr (D.B.); almamihaljevic12@gmail.com (A.M.P.); marija.bozicevic@kbc-zagreb.hr (M.B.); 2 School of Medicine, University of Zagreb, 10000 Zagreb, Croatia; 3 Laboratory for Molecular Neuropsychiatry, Division of Molecular Medicine, Ruder Boskovic Institute, 10000 Zagreb, Croatia; mnikolac@irb.hr; 4 Department of Laboratory Diagnostics, University Hospital Centre Zagreb, 10000 Zagreb, Croatia; ana.kozmar@kbc-zagreb.hr (A.K.); drogic@unizg.pharma.hr (D.R.); 5 Faculty of Pharmacy and Biochemistry, University of Zagreb, 10000 Zagreb, Croatia; 6 University of Applied Sciences Hrvatsko Zagorje Krapina, 49000 Krapina, Croatia

**Keywords:** bright light treatment (BLT), interleukin-6 (IL-6), treatment-resistant depression (TRD), female subjects

## Abstract

Treatment-resistant depression (TRD) remains a challenge despite the growing number of interventions. Peripheral interleukin-6 (IL-6) levels have repeatedly been associated with both the presence and response to different treatments in TRD. There is currently no information available on the effects of bright light therapy (BLT) on serum IL-6 levels. This study assessed the effects of BLT on serum IL-6 levels in TRD patients. Serum IL-6 was determined at two points in TRD patients—at baseline and after 4 weeks of BLT—and at a single point in the healthy controls. Depression severity was measured by the Hamilton Rating Scale for Depression (HAMD)-17 and the Montgomery–Åsberg Depression Rating Scale (MADRS). The study included 104 females, 54 diagnosed with TRD (median age 52.5) and 50 healthy controls (median age 44.5). At baseline, patients had higher IL-6 levels than the controls. BLT treatment reduced HAMD-17 and MADRS scores. Serum IL-6 levels were not significantly affected by the 4 weeks of BLT. However, when patients were divided according to treatment response, IL-6 levels were increased in responders to BLT. The neuroinflammatory mechanism may be involved in the etiopathogenesis and the treatment of TRD, while changes in serum IL-6 levels may be potential indicators of response to treatment.

## 1. Introduction

Depression is a prevalent and frequently severe psychiatric condition that can lead to significant impairment, with an aggregate lifetime prevalence of 10.8% in community samples [1]. While a global increase of 27.6% in cases of major depressive disorder (MDD) has been reported due to the COVID-19 pandemic [2], patients previously diagnosed with depression were also the most vulnerable group among psychiatric patients during the COVID-19 pandemics, and some other disasters such as earthquakes [3]. Antidepressants are the first-line medical intervention for MDD, but a significant number of patients with MDD do not show a response, even to numerous antidepressant regimens, resulting in difficult-to-treat depression called treatment-resistant depression (TRD) [4]. The prevalence of TRD among MDD patients varies between 11 and 56% across studies [5,6,7], and these differences are most likely induced with different methodologies and different clinical subpopulations.

While there is no convenient biomarker able to forecast a patient’s reaction to antidepressant therapy, numerous candidates have been proposed [8]. Inflammatory responses play an essential role in the intricate pathophysiology and neurobiological underpinning of MDD [9,10]. Namely, depressed patients exhibit elevated levels of the pro-inflammatory cytokine IL-6 compared to control participants [11], which was evident even in drug-naïve first-episode MDD participants [12]. It was proposed that normalizing IL-6 activity is essential for treating depression linked to inflammation [10].

Numerous treatment options have been used for the treatment of TRD patients, such as atypical antipsychotics, thyroid hormones, dopaminergic compounds, and lithium [13,14]. Electroconvulsive therapy (ECT) continues to be a highly effective therapeutic option for TRD [15], while evidence is accumulating regarding the rapid improvement of symptoms by ketamine and esketamine [16]. Within the increasing number of pharmacological and non-pharmacological alternatives for TRD, bright light therapy (BLT) is considered as a promising option, due to its accessibility, affordability, user-friendly procedure, and favorable tolerability [17]. Light exerts an antidepressant effect by stimulating retinal ganglion cells, which, through further neuronal pathways, affect the suprachiasmatic nucleus (SCN) and lateral habenula (LHb) cells [18]. In addition to antidepressant treatments, BLT demonstrated efficacy in TRD [17,19,20], whether used independently or as a combined treatment approach with other therapeutic methods [21,22,23].

IL-6 is the most often studied cytokine linked to TRD [24], and it is associated with the treatment effects. Namely, in the ketamine administration trial (in dosage of 0.5 mg/kg ketamine over 40 min), baseline serum IL-6 levels were found to be elevated in responders compared to both non-responders and controls (who had similar serum IL-6 values), suggesting that serum IL-6 serves as an effective indicator of a swift response to antidepressant treatment [25]. In contrast, among patients free from antidepressants for at least two weeks, serum IL-6 was decreased from 230 min to 3 days post-infusion in those who responded well, whereas it remained unchanged in non-responders [25]. In agreement, plasma IL-6 levels showed a reduction 26 days following the initiation of ketamine infusions [26] and after 6 esketamine applications [27], and IL-6 levels were associated with a reduction in symptoms of depression [26] but not with the changes in anhedonia scores [27]. More complex findings were reported in ECT treatment, which included an initial increase, consequent decrease, and eventually no change in IL-6 levels [28].

As far as we are aware, only two studies [29,30] and one review [10] reported on the influence of BLT on peripheral, i.e., plasma IL-6 levels in individuals diagnosed with depression [10,29,30]. Namely, plasma IL-6 levels showed no change in 14 patients with seasonal affective disorder (SAD) [29]. Another study [30] reported no differences in IL-6 levels between individuals with or without depression, but a correlation between the duration of sunlight exposure and plasma IL-6 levels was identified, showing that high amounts of light exposure were significantly related to higher IL-6 levels in individuals diagnosed with depression. Since the studies of BLT in patients with TRD are scarce [17], and due to the absence of studies exploring the relationship between serum IL-6 levels and BLT in this population, this study sought to measure serum IL-6 levels before and after 4 weeks of treatment with BLT in TRD.

## 2. Materials and Methods

### 2.1. Participants and Study Design

In this study, 54 female patients diagnosed with TRD who met the specified inclusion and exclusion criteria during the screening process were included. The recruitment of the participants diagnosed with TRD was carried out at the Department of Psychiatry and Psychological Medicine, University Hospital Center Zagreb as outpatients or as hospitalized patients. Participants signed the informed consent document. The study protocol was approved by the Ethics Committees of the University Hospital Center Zagreb and the Medical School of the University of Zagreb. Their serum IL-6 levels were compared to the serum IL-6 levels of healthy women as a control group.

According to the inclusion criteria, eligible female patients were aged from 20 to 70 years and had continued to take the same antidepressant therapy as prescribed by their psychiatrist for at least 4 weeks before the enrollment to the study. The diagnosis of MDD was verified through a structured clinical interview adhering to the DSM-5 criteria [31]. MDD can be considered as TRD as previously reported; TRD is defined as the inadequate response following two unsuccessful attempts with adequate duration and dosage of antidepressants from various chemical groups [32].

The exclusion criteria included psychiatric comorbidities including SAD and bipolar disorder (BD); suicidality; alcohol and other substance dependence; cranio-cerebral injury or surgery; somatic and neurological comorbidities such as malignancy, dementia, immune disease, epilepsy, cerebral aneurysm, acute infectious diseases, allergic reaction, serious ocular conditions like glaucoma, retinopathy, and eye infections; the use of ECT within three months prior to the study; participation in individual psychotherapy; prior utilization of BLT; pregnancy; and breastfeeding. The use of antipsychotics and mood stabilizers was not permitted. Anxiolytics and short usual psychotherapeutic support were allowed as an essential segment of the psychiatric examination. 

The severity of depression in patients was evaluated using the Montgomery–Åsberg Depression Rating Scale (MADRS) [33] and the Hamilton Depression Rating Scale-17 (HAMD-17) scores [34] at inclusion and after 4 weeks of BLT. The inclusion and exclusion criteria are the same as previously reported [35]. Therefore, this study presents a secondary analysis of the obtained data regarding the effect of BLT on BDNF levels in plasma of patients with TRD [35]. Definition of clinical response was a ≥50% reduction from baseline in the HAMD-17 or MADRS scores [36,37] following a duration of four weeks of BLT. Blood was taken in a test tube at 8:30 a.m. after fasting, following the same protocol as at baseline and after 4 weeks of BLT.

In addition, a control group of 50 healthy age-matched women was sampled with the similar protocol, and blood was taken in a test tube at 8:30 a.m., but only once, to assess their serum IL-6 levels.

The study procedures are presented in Table 1, and the protocol was the same as in our previous study [35].

### 2.2. Bright Light Therapy

A psychiatrist performed the BLT using a *Bioptron Pro 1* lamp manufactured by Zepter, Wollerau, Switzerland, light intensity 10,000 lx, careful to leave a space from 30 to 40 cm between the lamp and the eyes. As described before [35], this lamp is a sophisticated device meant only for professional medical use. The *Bioptron Pro 1* lamp produces light that closely resembles the portion of the electromagnetic spectrum that is naturally emitted by the Sun, but it is devoid of UV radiation [38,39]. The protocol of BLT was followed as previously described [35]; BLT was administered for 20 min in the morning as an additional therapy, according to the program, 4 days a week and for 4 weeks.

### 2.3. Blood Sampling and Serum IL-6 Levels Measurement

Blood samples were taken after fasting during the night, at 8.30 a.m., during regular laboratory appointments. Samples were collected in standardized 4.5 mL negative pressure tubes with a clotting activator (Greiner Bio-One International AG, Kremsmünster, Austria). The samples then were centrifuged for 10 min at 3000 rpm (Hettich Rotofix 32 A; Andreas Hetich GmbH&Co. KG, Tuttlingen, Germany). The obtained serum was distributed into plastic 1.5 mL test tubes (Kartell, Noviglio, Italy), which were then stored at −80 °C until the test was performed.

Serum IL-6 levels were determined using a commercial enzyme-linked immunosorbent assay (ELISA). The tests were performed following the detailed instructions of the manufacturer (Invitrogen, Thermo Fisher Scientific Inc., Vienna, Austria).

In performing the ELISA test, 50 μL of diluent and then 50 μL of controls or undiluted serum samples were added to the wells of microtiter ELISA plates. Wells were coated with a specific human IL-6 monoclonal antibody, and 100 μL of previously diluted standard samples were added to separate wells. To all wells, 50 μL of monoclonal anti-human IL-6 antibody conjugated with biotin was added after that step. After incubation on a shaker for two hours at room temperature, the plate was washed six times with washing solution. Later, 100 μL of Streptavidin-HRP conjugate was added to each well, followed by incubation for one hour on a shaker at room temperature. After washing and adding 100 μL of the prepared amplification reagent I (Biotinyl-Tyramide), the wells were incubated for 15 min on a shaker. The plate was washed again six times, and 100 μL of amplification reagent II (Streptavidin-HRP) was added to all wells and incubated on a shaker for 30 min. After the last washing and with the addition of 100 μL of tetramethyl benzidine substrate solution, the incubation lasted for 20 min, with the wells protected from light. Adding 100 μL of 1 M phosphoric acid stopped the reaction. The resulting developed color was read as absorbance at 450 nm with a reference wavelength of 620 nm on a Sunrise microtiter plate reader (Tecan Trading AG, Männedorf, Switzerland). The measurement results in pg/mL were calculated according to the standard curve (Magellan software, Tecan Trading AG, Männedorf, Switzerland). The standard curve was derived from the known concentrations of IL-6 in the serum standards.

### 2.4. Data Analysis/Statistics

The SAS software, version 9.4M6 from 2018 (SAS Institute, Cary, NC, USA) was used for data analysis and statistical evaluation. All used statistical tests were two-tailed, and the α significance level was set at 0.05. The normality or escape of the normal distribution for all analyzed variables was assessed utilizing the Kolmogorov–Smirnov test. The median and the range (min–max value) values are presented for all clinical and sociodemographic variables, and IL-6 serum concentration, as they deviated from the normal distribution. For two independent groups, the Mann–Whitney U test was used, while for the comparison of three or more independent groups, Kruskal–Wallis ANOVA by ranks was used. The Wilcoxon rank test of dependent samples (Wilcoxon test) was used for the comparison of two dependent groups. To evaluate the potential impact of age on serum IL-6 concentration, multiple linear regression analysis was used in patients with TRD and healthy controls. The Spearman correlation coefficient was used to evaluate correlation between age and serum IL-6. Analysis of covariance (ANCOVA) was used to compare IL-6 serum concentration after BLT between groups of participants subdivided according to their response to BLT, or for those who achieved remission. These comparisons were made using the initial concentration of serum IL-6 as a covariate to correct the results. Prior to ANCOVA, log10 data transformation of IL-6 serum concentration was performed due to the deviation from normal distribution. The fold change (FC) in IL-6 serum concentration was calculated as the ratio (B/A) of the IL-6 serum concentration following a four-week period of BLT (B) and IL-6 serum concentration at baseline (A).

## 3. Results

### 3.1. Subject Characteristics

This study included a total of 104 female subjects, 54 diagnosed with TRD and 50 healthy controls. Participants differed significantly in age (U = 831.00; *p* = 0.003) since subjects diagnosed with TRD (median = 52.5; range = 21–66) were older than the healthy controls (median = 44.5; range = 23–65). To assess the potential impact of age on serum IL-6 concentration, multiple linear regression analysis was used in patients with TRD and healthy controls. Serum IL-6 levels were used as the dependent variable, while age and diagnosis were set as the independent variables. The generated model was not significant (F = 2.94; df = 2, 97; *p* = 0.117; Radj2 = 0.024) and suggested there was no significant association between age (*p* = 0.183) and serum IL-6 levels. We additionally evaluated the correlation between age and serum IL-6 levels in both subject groups. The results suggested no correlation between the two variables when evaluating all subjects together (rs = 0.142; *p* = 0.160), or when separately evaluating TRD subjects (rs = −0.052; *p* = 0.722) and healthy controls (rs = 0.178; *p* = 0.215).

### 3.2. Serum IL-6 Concentration Before BLT

**Figure 1:**
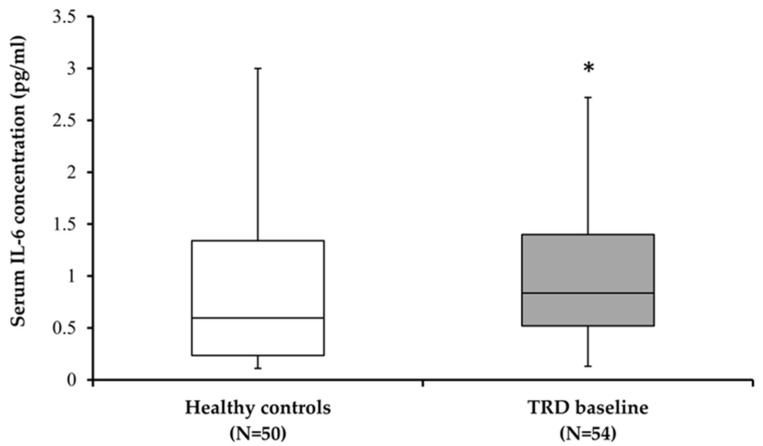
Serum IL-6 levels in healthy subjects and subjects with treatment resistant depression (TRD) before BLT. * *p* = 0.032 vs. healthy female control subjects (Mann–Whitey test).

After excluding age as a possible covariable, serum IL-6 levels were compared between subjects diagnosed with TRD and healthy control subjects (U = 938.50; *p* = 0.032). The significant difference was due to the higher serum IL-6 levels in patients with TRD than in the healthy controls (Figure 1).

### 3.3. Effect of BLT on Serum IL-6 Concentration

**Figure 2:**
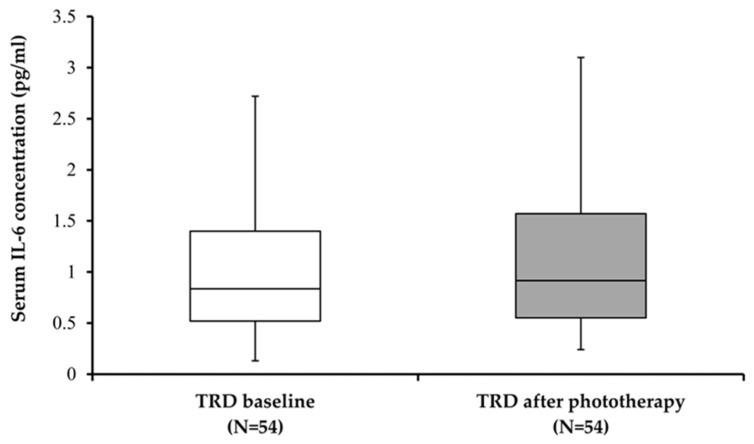
Serum IL-6 levels in subjects with treatment-resistant depression (TRD) before and after 4 weeks of BLT.

In all patients with TRD, serum IL-6 levels were assessed for the possible effect of BLT before and after four weeks of treatment. Possible influence of BLT on serum IL-6 levels was evaluated with Wilcoxon test, which revealed that serum IL-6 levels did not differ significantly before and after BLT (Figure 2; W = 580.00; *p* = 0.746).

**Figure 3:**
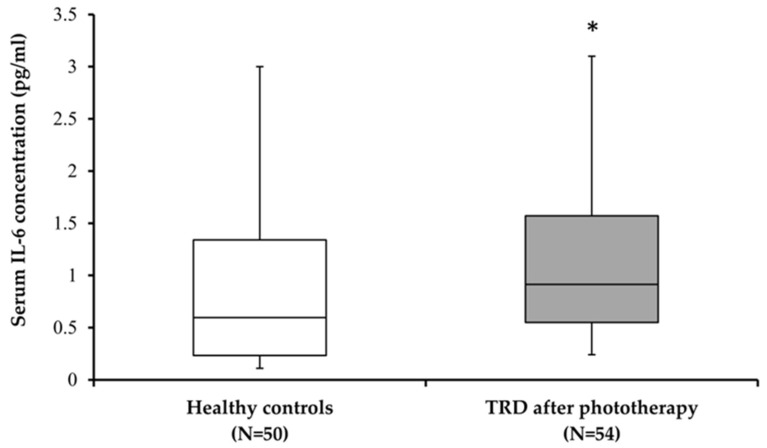
Serum IL-6 levels in healthy subjects and subjects with treatment-resistant depression (TRD) after 4 weeks of BLT. * *p* = 0.022 vs. healthy female control subjects (Mann–Whitey test).

We additionally compared IL-6 serum levels between healthy controls and MDD subjects after 4 weeks of BLT (Figure 3). The concentration of serum IL-6 was significantly increased following a four-week period of BLT in patients with TRD when compared to healthy controls (Figure 3; U = 917.00; *p* = 0.022).

Results showing median HAMD-17 and MADRS scores and IL-6 levels, before and following a four-week period of BLT in TRD patients, are presented in Table 2.

The HAMD-17 and MADRS scores were significantly (*p* < 0.001) lower after BLT compared to the baseline scores (Table 2), while there were no significant differences in serum IL-6 levels following a four-week period of BLT compared to the baseline values in TRD patients (Table 2, Figure 2).

In order to determine a potential association between the altered IL-6 serum level after BLT and the improvement in depression symptoms, participants were divided into those who responded well, i.e., responders, and those who did not respond well, i.e., non-responders, to BLT. Response was defined as a ≥50% reduction in depression severity after treatment, evaluated by the HAMD-17 and/or MADRS scores. The fold change in serum IL-6 levels and the fold change in HAMD-17 and MADRS scores were calculated as the ratio (B/A) of the values of these parameters after BLT (B) and the values before BLT (A). The comparison was made using ANCOVA and the initial concentration of serum IL-6 was used as a covariate to correct the results. Prior to ANCOVA, a log10 transformation was performed to achieve normal distribution for all the variables in the equation. The ANCOVA results showed significant (*p* = 0.041) difference in IL-6 concentration fold change in subjects who responded well to BLT (HAMD-17 scores decrease ≥ 50%) compared to those who were identified as non-responders (HAMD-17 scale scores decrease <50%) (F = 15.14; df = 2, 51; *p* < 0.001). Namely, responders had significantly higher serum IL-6 levels than non-responders (Table 3).

A similar effect was detected when TRD subjects were subdivided with regard to the change in MADRS scores (F = 18.35; df = 2, 51; *p* < 0.001) (Table 4). There were significant differences (*p* = 0.006) in IL-6 concentration fold change in subjects who responded well to BLT (MADRS scores decrease by ≥ 50%) compared to non-responders, which was due to the significantly increased serum IL-6 levels in responders compared to non-responders (Table 4).

## 4. Discussion and Conclusions

The study results revealed higher serum IL-6 levels in female TRD patients in relation to healthy female subjects, and increased IL-6 levels in responders to BLT compared to non-responders. The elevated serum IL-6 levels in female patients with TRD in relation to female healthy controls are in line with other findings, showing increased IL-6 levels in TRD patients [40,41], although unchanged IL-6 levels were also detected in TRD [42]. Besides TRD patients, depressive patients had higher peripheral IL-6 levels compared to healthy controls [43,44], but after various combined antidepressant treatments, IL-6 levels were mostly decreased [14]. This meta-analysis revealed that non-responders had increased plasma IL-6 levels, and the majority of the different treatment strategies seem to reduce IL-6 levels and improve symptoms of depression [14].

In the present study, we were not able to identify significant differences in serum IL-6 levels before and after BLT in TRD subjects, while IL-6 levels slightly increased after 4 weeks of BLT; therefore, the difference compared to healthy controls was even more pronounced. However, the important finding was a significant difference in IL-6 levels fold change in responders to BLT compared to non-responders, according to the HAMD-17 and MADRS scores, since ANCOVA revealed elevated IL-6 levels in patients with TRD who responded well to BLT. The findings indicated a notable distinction between responders and non-responders to BLT, as measured by both HAMD-17 and MADRS scores. This difference was characterized by a more pronounced increase in serum IL-6 levels among those who responded positively to BLT, in contrast to individuals who exhibited a poorer response (non-responders). This research appears to be the first of its kind, to the best of our knowledge, to measure IL-6 after BLT in TRD patients, and therefore we could not compare our results with data from the literature. The sample size, i.e., the number of female participants with TRD (N = 54) treated with BLT, and the 50 female subjects in the healthy control group included in the present study align with the numbers of depressive subjects (both MDD and TRD) who participated in the previous studies evaluating different combined anti-depressive treatment strategies and IL-6 levels [14], supporting the strength of this study.

Although numerous studies have examined the effect of certain antidepressant strategies on inflammatory parameter IL-6 [14], different treatments resulted in diverse effects on IL-6. Many studies reported that antidepressants decrease peripheral IL-6 levels [14,44,45], but the findings are still inconsistent when evaluating associations between different antidepressant treatments and serum IL-6 levels [14].

There were only two studies evaluating IL-6 levels in depressed patients following the use of light therapy [29,30]. These studies described the improvement of symptoms in 64% of the 15 subjects with SAD after 2 weeks of BLT. However, there was no observed impact on plasma IL-6 levels [29], nor were there any differences in IL-6 levels between those with and without depression [30]. The length of exposure to daylight in Brazil did not change plasma IL-6 levels in healthy individuals, while shorter exposure to daylight decreased plasma IL-6 levels, and longer exposure to daylight increased plasma IL-6 levels in subjects diagnosed with depression [30].

These results were explained by the dysfunction of the SCN and the irregularity in melatonin secretion that underlie these IL-6 variations [30]. Melatonin, as an endogenous synchronizer, coordinates the circadian rhythm of sleep and wakefulness, as well as the secretion of cytokines, which also show diurnal variations [46,47].

Other findings related to different disorders treated with light therapy and IL-6 levels are as follows: Blue light phototherapy effectively regulated neuroinflammatory responses and reduced symptoms in subjects who developed depression after stroke, and plasma IL-6 levels in the experimental group were found to be reduced following 8 weeks of daily 30 min blue light phototherapy compared to the control group [48]. In individuals suffering from immune-metabolic depression (IMD) and type 2 diabetes mellitus (T2DM), randomized for 4 weeks to a broad-spectrum daily BLT group of 10,000 lx for 30 min and to a placebo group, BLT had no effect on changes in serum IL-6 levels [49]. The treatment for 4 weeks using a standard regimen of BLT, which involved a 10,000 lx fluorescent white light box equipped with an ultraviolet filter, was administered at home for 30 to 60 min each morning. This approach resulted in a reduction in various pro-inflammatory cytokines, including interleukin (IL)-1β, tumor necrosis factor (TNF)-α, and interferon (IFN)-γ, in patients with SAD [50], but IL-6 was not measured.

Other therapeutic strategies used to treat TRD are as follows: Ketamine infusion (4 h) decreased serum IL-6 levels [40]. The initial findings indicate that elevated serum IL-6 levels could serve as a biological marker for a more favorable therapeutic response in TRD (25). ECT resulted in elevated plasma IL-6 levels [51], while serum IL-6 levels remained unchanged [52]. As ECT was reported to increase plasma IL-6 levels but does not affect serum IL-6 levels, it might be speculated that BLT has a similar effect on IL-6, but studies evaluating the influence of BLT on serum and plasma IL-6 levels in depressed subjects are missing. Namely, IL-6 levels were increased 4 h after the application of ECT but returned from its elevated basic values to normal levels only in remitters according to the MADRS [53]. In another study [54], IL-6 levels increased further from the initial to the second measurement, before the third ECT, but decreased within a week after the ECT completion. Therefore, these results suggest that ECT affects peripheral IL-6 levels, indicating a relationship with a transient inflammatory reaction, achieving a therapeutic effect [53]. A study involving eight patients with TRD who underwent invasive deep brain stimulation (DBS) provides support for this hypothesis, in which a local inflammatory reaction and a rapid antidepressant response occurred shortly after implantation, while anti-inflammatory drugs led to a recurrence of depressive symptoms [55].

Inflammation, stress, and depression are interconnected. IL-6 acts via IL-6 receptors (IL-6Rs). After binding to the target cell, there are two signaling pathways—anti-inflammatory and pro-inflammatory [56]. As opposed to classical anti-inflammatory signaling in peripheral cells, in the brain, IL-6 acts on different mood circuits, affects different neurotransmitters, has pleiotropic effects, and engages in pro-inflammatory trans-signaling pathways [56]. IL-6 is a cytokine with multiple roles in acute inflammation, the activation of osteoclasts, and the proliferation and differentiation of B lymphocytes [57]. In the etiology of many physical diseases, such as diabetes, malignancies, and autoimmune conditions, IL-6 acts as a pro-inflammatory cytokine that enhances and maintains the inflammatory state [58]. Similarly, MDD patients have consistently been observed to exhibit elevated levels of IL-6 [14]. For the treatment of TRD, our present and previous [35] studies confirm BLT is an effective therapy. This result is in line with data from other small studies where BLT was found to be efficacious in TRD [19,20].

The findings from our BLT study reveal a notable variation in the increase in IL-6 concentration between the two groups of TRD patients with regard to the clinical response to BLT, as responders had higher IL-6 levels than non-responders. This suggests that elevated peripheral IL-6 levels may be linked to an initial inflammatory response for achieving an antidepressant effect, and this hypothesis should be further investigated. Moreover, further studies are warranted to explore the connection between light therapy and the change in the inflammatory reaction as part of the mechanism of action of light therapy in more detail. To test this hypothesis, we propose the exposure to bright light of a 10,000 lx intensity for at least 8 weeks, with the participation of a larger number of subjects (both males and females), and measurements of peripheral IL-6 and other significant cytokines at multiple time points.

Due to ethical reasons, the healthy control group was not exposed to a BLT, and this might be a limitation of this study.

In conclusion, the neuroinflammatory mechanism could be of great importance in the etiopathogenesis and treatment of TRD, while changes in serum IL-6 levels might be used as potential indicators of treatment response. As IL-6 acts in the periphery, as well as in the brain, and plays an important role in depression, studies on IL-6 might help in the development of novel potential therapeutic strategies targeting/blocking Il-6 or its receptors in the periphery to treat depression [59]. This should be further verified by future clinical studies. In addition, BLT, recognized for its safety and convenience, may play a significant role in the treatment of TRD.

## Figures and Tables

**Table 1:** Study procedures.

	TRD	Group	Control Group
	Baseline (T0)	4 weeks of BLT (T1)	
Structured clinical interview, clinical and demographic data	x		
HAMD-17, MADRS	x	x	
Serum IL-6 concentration	x	x	x

HAMD-17: Hamilton Depression Rating Scale-17; MARDS: Montgomery–Åsberg Depression Rating Scale; IL-6: interleukin-6; TRD: treatment-resistant depression; BLT: bright light therapy.

**Table 2:** Results of the median HAMD-17 and MADRS scores and IL-6 concentration, before and after 4 weeks of BLT in patients with TRD.

Parameters		Median (Min–Max)	Wilcoxon Signed-Rank Test	
			W	*p*
HAMD-17	Baseline	21 (16–17)	0.00	<0.001
	After BLT	10 (3–17)		
MADRS	Baseline	27 (19–36)	0.00	<0.001
	After BLT	13 (4–21)		
IL-6 (pg/mL)	Baseline	0.88 (0.13–9.66)	679.00	0.747
	After BLT	1.02 (0.24–4.57)		

HAMD-17: Hamilton Depression Rating Scale-17; MARDS: Montgomery–Åsberg Depression Rating Scale; IL-6: interleukin-6; BLT: bright light therapy.

**Table 3:** Serum IL-6 levels in responders and non-responders according to the HAMD-17 rating scale before and after 4 weeks of BLT.

HAMD-17		Responders (N = 29)	Non-Responders (N = 25)	Mann–Whitney U Test	
		Median (Min–Max)		U	*p*
IL-6 (pg/mL)	Baseline	0.77 (0.13–6.51)	1.32 (0.42–9.66)	431.50	0.231
	After BLT	1.06 (0.31–4.57)	0.99 (0.24–2.74)	329.50	0.567
	FC	1.27 (0.38–4.71)	0.79 (0.10–2.21)	245.00	0.041

HAMD-17: Hamilton Depression Rating Scale-17; IL-6: interleukin-6; FC: fold change; BLT: bright light therapy.

**Table 4:** Serum IL-6 levels in responders and non-responders according to the MADRS before and after 4 weeks of BLT.

MADRS		Responders (N = 28)	Non-Responders (N = 26)	Mann–Whitney U Test	
		Median (Min–Max)		U	*p*
IL-6 (pg/mL)	Baseline	0.78 (0.13–6.51)	0.92 (0.42–9.66)	427.00	0.275
	After BLT	1.11 (0.37–4.57)	0.90 (0.24–2.74)	289.50	0.197
	FC	1.28 (0.38–4.71)	0.76 (0.10–1.98)	206.50	0.006

MARDS: Montgomery–Åsberg Depression Rating Scale; IL-6: interleukin-6; FC: fold change; BLT: bright light therapy.

## Data Availability

Data are unavailable due to privacy or ethical restrictions.

## Abbreviations

The following abbreviations are used in this manuscript:

MDD	Major depressive disorder
TRD	Treatment-resistant depression
BLT	Bright light therapy
IL-6	Interleukin-6
SCN	Suprachiasmatic nucleus
SAD	Seasonal affective disorder
ECT	Electroconvulsive therapy
